# Development and validation of a tool for the assessment of benefit from treatment of allergic rhinitis in children and adolescents (PBI-AR-K)

**DOI:** 10.1186/s13223-022-00733-8

**Published:** 2022-10-25

**Authors:** Toni Maria Janke, Elisabeth Eisner, Matthias Augustin, Christine Blome

**Affiliations:** Competence Center for Health Services Research in Dermatology (CVderm), Institute for Health Services Research in Dermatology and Nursing (IVDP), University Medical Center Hamburg-Eppendorf (UKE), 20246 Hamburg, Germany

**Keywords:** Patient-relevant benefit, Treatment goals, Children, Adolescents, Patient-reported outcomes

## Abstract

**Background:**

Allergic rhinitis (AR) is frequent in children and adolescents and can severely affect their lives. This article describes the development and validation of a questionnaire to assess treatment needs and benefits in children and adolescents, the PBI-AR-K, in a sample of patients receiving grass pollen sublingual immunotherapy.

**Patients and methods:**

The PBI-AR-K was developed based on an open survey including children and adolescents and expert consensus between methodologists, patients, and physicians. The PBI-AR-K assesses patient needs before the treatment and perceived benefit during or at the end of a treatment. A weighted benefit score can be calculated ranging from 0 to 4 (4 = highest possible benefit). The validation was conducted in children (5–12 years) and adolescents (13–17 years) receiving sublingual immunotherapy. Subscales were developed based on factor analysis. Psychometric properties of items and scales were assessed with descriptive statistics, internal consistency, and convergent validity.

**Results:**

The final PBI-AR-K consists of 19 items. For validation, data from 345 patients (mean age 11.1; 60.9% male; n = 223 children; n = 122 adolescents) was analysed. Factor analyses resulted in four subscales for children and three subscales for adolescents. The items with the highest importance ratings were about choice of leisure activities (mean value in children: 3.5) and about being free of AR symptoms (adolescents: 3.3). The weighted PBI-AR-K scores reflected considerable patient-reported benefit (2.08–2.82) in both children and adolescents. Internal consistency of all scales was good or acceptable. In the children’s sample, the global scale and three of four subscales were quite consistently correlated with convergent variables, while the subscale ‘treatment burden’ was significantly correlated only with change in average impairments due to rhinitis symptoms. The adolescents’ sample showed more inconsistent results with only change in rhinitis severity being significantly associate with all subscales.

**Conclusion:**

The newly developed PBI-AR-K is a reliable and valid questionnaire for use in children; for the use in adolescents, it should be further elaborated.

## Introduction

The prevalence of allergic rhinitis (AR) and allergic rhinoconjunctivitis (ARC) increases throughout childhood, peaking in teenage years [1, 2]. AR is an immunoglobulin E-mediated allergic reaction [3], manifesting in sneezing, nasal congestion, clear rhinorrhoea, and nasal or palatal pruritus [4]. ARC (i.e., the simultaneous appearance of nasal and ocular allergy symptoms) is prevalent in 8.5% of 6- to 7-year-olds and in 14.6% of 13- to 14-year-olds worldwide, with large regional variations [1]. AR is more prevalent in girls than in boys throughout childhood, whereas in adolescence more boys than girls are affected [5, 6].

AR can be classified according to its severity (mild vs. moderate to severe) and its patterns of occurrence (persistent vs. intermittent) [4]. Moderate to severe forms and persistent forms of AR have particularly great impact on adolescents’ and adults’ health-related quality of life (HRQoL), affecting sleep, daily activities, and school/work performance [7]. In comparison to adults, children report more severe forms of AR and more concomitant asthma, conjunctivitis, and atopic dermatitis [8]. This is one of the reasons why the results of studies with adults may not be directly transferable to younger populations.

Treatment options for AR are the avoidance of allergens and pharmacotherapeutic interventions, both of which aim to reduce symptoms. The only treatment option being able to alter the course of AR is allergen immunotherapy (AIT), which provides short-term as well as long-term benefits in the treatment of AR [9].

Besides clinical efficacy, patient-reported benefits are decisive outcomes for evaluating a treatment. Achieving patient-relevant benefits is a therapy goal in itself; beyond that, it can enhance treatment satisfaction and, hence, patient adherence [10], which is important for clinical efficacy [11]. Patient-reported outcome measurements, especially HRQoL questionnaires, have gained importance to assess treatment benefits from the patient's perspective. However, the relevance of different benefits will differ according to patients’ individual needs and expectations, which are not considered in most HRQoL questionnaires. Furthermore, benefit assessment via HRQoL is based on comparison of pre- and post-values, an approach that can be susceptible to response shift bias [12]. Such bias can be avoided by direct benefit assessment after treatment. This approach is implemented in the Patient Benefit Index (PBI), a tool to measure patient-relevant treatment benefits, taking into account patients' needs and expectations. In general, patients’ needs go well beyond the pure reduction of symptoms [13]; they can be assigned to dimensions such as ‘psychological burden’, ‘treatment burden’, ‘physical burden’, and ‘activity/physical capability’ [14].

PBI questionnaires have been developed, standardised, and validated for a wide range of indications, including AR (PBI-AR) [13] and skin diseases (e.g., Augustin et al. 2009, for different skin diseases [15]; Feuerhahn et al., for psoriasis [16]; Augustin et al. 2012, for chronic wounds [17]; Blome et al. 2009, for pruritus [18]), but mostly for adult populations. Using measurement instruments designed for adults in paediatric and adolescent populations is not suitable as specific dimensions of patient-relevant constructs and their operationalisation vary depending on the respondents’ age [19]. Additionally, questionnaires designed for adults may contain language difficult to understand for younger patients and may be burdensome due to their length [20]. Accordingly, age-appropriate questionnaires are required to obtain valid and reliable data [21]. Moreover, ARC-specific disease burden and impairments experienced by younger patients differ from those of adults [22, 23], which requires adapted questionnaires for this specific condition.

This article describes the development and evaluation of psychometric properties of a PBI questionnaire in German language specifically designed to assess treatment needs and benefits in children and adolescents with AR, the PBI-AR-K (K for kids).

## Methods

### Patient Benefit Index

The PBI-AR has been previously developed and validated for the use in adults and contains 25 items [13]. Each PBI has two parts: The Patient Needs Questionnaire (PNQ), which is completed at the beginning of a treatment, and the Patient Benefit Questionnaire (PBQ), which is completed during or at the end of a treatment. Both parts consist of the same items, with the PNQ asking the patients to rate the importance of each item and the PBQ asking to rate the achievement of the respective item. Patients answer all items on a 5-point Likert scale (’not at all’ to ‘very’) and, alternatively, have the option to state ‘does/did not apply to me’. From all items, a weighted index value is calculated by multiplying the achieved benefit of each item (PBQ) by its importance (PNQ), dividing it by the sum of the importance of all items, and summing up the resulting quotients of all items. This total score can range from 0 to 4, with higher scores indicating higher benefits. Scores ≥ 1 are considered relevant.

### Development of the PBI-AR-K

#### Open survey

In the first step of developing the PBI-AR-K, an open patient survey was conducted. Children and adolescents with physician-confirmed AR and being aged 5 to 17 years were recruited in two federal states in Germany (Hamburg and Schleswig–Holstein). Participants completed a questionnaire with open questions about their most important needs and therapy goals themselves or, if they felt not fluent enough in reading and writing, their parents read out the questionnaire to them and noted their answers.

#### Expert discussion

All patient statements from the open survey were discussed in an expert panel including each one methodologists, patients, and physicians. This group categorised all patient statements qualitatively and phrased items thereupon, using easy language to facilitate readability and comprehensibility for children and adolescents. If language and content were appropriate, the same wording as in the adult PBI-AR was used. The wording of the instruction was adapted to the target population in discussions involving teachers, psychologists, and physicians.

### Validation of the PBI-AR-K

#### Participants

Psychometric properties of the PBI-AR-K were evaluated as part of a non-interventional study on benefits of sublingual immunotherapy (SLIT) [24]. In this study, patients received Oralair, a 5-grass pollen sublingual tablet in real-life practice. Children (5 to 12 years), adolescents (13 to 17 years), and adults (18 years and older) were included with adults answering the PBI-AR, and children and adolescents answering the PBI-AR-K. Sample size calculation was fitted for the observational study of the SLIT, not for the validation of the PBI-AR-K, which is more than adequate based on sample size suggestions for validation [25]. For the purpose of this article (i.e., the validation of the PBI-AR-K), only data from children and adolescents are presented. Patients were recruited consecutively by a nationwide sample of 145 allergologically experienced physicians across Germany. Decision for SLIT had been made prior to study inclusion and independent from this non-interventional study. Inclusion criteria were being aged 5 or older and having a grass-pollen and/or rye pollen or cereal pollen induced ARC. Exclusion criteria were the contraindications for the treatment (i.e., severe and/or unstable asthma, severe immune deficiency or auto-immune disease, malignant disease, oral inflammations, hypersensitivity to any of the excipients). Patients (and their parents/legal representatives for patients < 18 years) gave written informed consent. Ethical approval was obtained from the Freiburg Ethics Commission International (012*/*1889).

#### Procedure

Recruitment took place over two periods. Each patient was observed during his/her first treatment year (i.e., from the first exposure to SLIT until the end of the first grass pollen season during which the patient was treated with SLIT), with two visits during the observation period. Visit 1 (study initiation visit) took place at the time of treatment initiation (fall/winter 2012/13 or fall/winter 2013/14). Visit 2 (study end visit) took place after the end of the respective grass pollen season (2013 or 2014). At both visits, patients completed paper-based questionnaires and physicians completed an electronic case report form. Adolescents aged 13 to 17 years completed the questionnaires on their own, whereas for children aged 5 to 12 years, parents read out the questionnaires and filled in the children’s answers. Table 1 shows the data that were assessed in this study.

**Table 1:** Sociodemographic data, clinical data, and convergent variables assessed from patients in the validation study

Data	Assessed by patients		Assessed by physicians		Response options
	V 1	V 2	V 1	V 2	
Sociodemographic data					
Age			X		Number
Sex			X		Male/female
Clinical data					
Presence of type-1-allergies (grass pollen, rye pollen, other cereals pollen)			X		Yes/no per option
Anamnesis: clinical manifestations (rhinitis, conjunctivitis, asthma, neurodermatitis / atopic eczema)			X		Yes/no per option
Anamnesis: concomitant allergies (none, birch, other trees, weeds, house dust mites, animal hair, moulds, others)			X		Yes/no per option
Convergent variables					
Global judgement of patients’ well-being (during the first grass pollen season on AIT) compared to the previous pollen season (before AIT)		X		X	Much better / somewhat better / unchanged / worse
Severity of rhinitis symptoms			X	X	None/mild/moderate/strong
Severity of conjunctivitis symptoms			X	X	None/mild/moderate/strong
Frequency of rhinitis symptoms			X	X	< 4 days per week / ≥ 4 days per week
Duration of rhinitis symptoms			X	X	< 4 consecutive weeks / ≥ 4 consecutive weeks
Effects on sleep			X	X	Normal/impaired
Effects on activities			X	X	Normal/impaired
Effects on performance			X	X	Normal/impaired
Average impairment due to rhinitis symptoms	X	X			Not at all disturbing (0) to extremely disturbing (10) (numeric verbal visual analogue scale)
Peak impairment due to rhinitis symptoms	X	X			Not at all disturbing (0) to extremely disturbing (10) (numeric verbal visual analogue scale)

*AIT* allergen immunotherapy

#### Statistical analysis

Means, standard deviations, medians, ranges, and frequencies were calculated to describe sample characteristics and distribution of PBI items and scales. In order to develop subscales, factor analyses with varimax rotation were conducted using the PNQ items. Internal consistency was determined using Cronbach’s alpha based on PNQ items. For convergent validity analysis, we determined the association of PBI-AR-K global and subscale scores with physicians’ and patients’ overall judgement of patients’ well-being (during the first grass pollen season on SLIT) compared to the previous pollen season (before SLIT), change in rhinitis and conjunctivitis severity, change in frequency and duration of rhinitis symptoms, changes in impaired sleep, impaired activities, and impaired performance, change in average impairment due to rhinitis symptoms, and change in peak impairment due to rhinitis symptoms using t-tests, analysis of variance (ANOVA), and Pearson correlation (r) according to the scale level of the data. Results of the t-tests were reported for either equal or inequal variance according to Levene’s tests, with significance levels lower than 0.1 indicating inequal variance [26]. For t-tests and ANOVAs, effect sizes were computed using Cohen’s d and eta squared (η^2^). Effect sizes were assumed to indicate small, medium, and strong effects when d = 0.2, d = 0.5, and d = 0.8, respectively, when η^2^ = 0.01, η^2^ = 0.06, and η^2^ = 0.14, respectively, and when r = 0.1, r = 0.3, and r = 0.5, respectively [27]. Analyses were performed using SPSS version 22 for Windows (IBM, Armonk, NY, U.S.). The significance level was determined at p = 0.05.

## Results

### Development of the PBI-AR-K

The open survey was completed by 11 children and adolescents with AR aged between 5 and 17 years. A subsequent expert panel (two physicians, methodologists and patients each) categorised all statements and developed items. This resulted in the PBI-AR-K encompassing 19 items. Of these, five items had the same wording and ten had the same content as the PBI-AR for adults. Four items were added (‘feel well even with having hay fever’, ‘feel more comfortable being around other people’, ‘not be excluded by others’, ‘have a comfortable treatment’). All items of the final PBI-AR-K can be seen in Tables 2 and 3. As this study was conducted in German, we applied a standardised translation process to be able to display the items in English language in this article. This included two forward- and two backward-translations, a consensus meeting with the professional translators conducting the forward translation, and a final proof-read by another translator.

**Table 2:** Descriptive statistics for Patient Needs Questionnaire

Item (treatment goal)		Children			Adolescents		
As a result of therapy, how important is it for you to …		N	Mean (SD)^a^	Quite/ very^b^ (%)	N	Mean (SD)^a^	Quite/ very^b^ (%)
1)	… no longer have to sneeze	214	2.70 (1.27)	61.7	116	2.76 (1.15)	57.8
2)	… no longer have a runny or stuffy nose	214	3.36 (0.96)	84.1	117	3.01 (1.09)	69.2
3)	… be able to breathe through your nose freely	214	3.37 (1.00)	84.1	115	3.13 (1.16)	77.4
4)	… feel less tired or groggy	215	2.52 (1.51)	61.9	115	2.43 (1.50)	60.0
5)	… be able to stay outdoors without symptoms	215	3.34 (1.14)	83.7	115	3.07 (1.15)	75.7
6)	… have an easily applicable treatment	211	3.28 (0.99)	82.0	113	3.01 (1.02)	71.7
7)	… not have itching eyes, nose or throat anymore	213	3.38 (1.04)	86.4	117	3.09 (1.19)	76.1
8)	… not have burning or watery eyes anymore	214	3.17 (1.29)	78.0	117	3.03 (1.35)	76.1
9)	… no longer have hay fever symptoms	213	3.51 (0.84)	89.7	117	3.33 (0.90)	86.3
10)	… be able to sleep better	215	2.64 (1.50)	65.1	117	2.39 (1.54)	55.6
11)	… need less time for treatment	211	2.90 (1.29)	67.3	117	2.66 (1.16)	61.5
12)	… feel well even with having hay fever	212	3.48 (0.82)	90.1	116	3.17 (1.05)	77.6
13)	… be able to do anything you want in your free time even while having hay fever	215	3.52 (0.96)	88.8	117	3.28 (1.11)	83.8
14)	… feel more comfortable being around other people	215	3.04 (1.35)	75.3	117	2.57 (1.44)	62.4
15)	… be focused at school	214	2.77 (1.54)	69.6	116	2.61 (1.49)	62.9
16)	… not be excluded by others	214	1.92 (1.82)	46.3	115	1.43 (1.63)	32.2
17)	… not to have to go to the doctor so often	215	2.99 (1.22)	71.2	115	2.67 (1.29)	60.9
18)	… have a comfortable treatment	215	3.28 (1.04)	81.4	117	3.05 (1.02)	73.5
19)	… have fewer side effects	214	3.19 (1.26)	79.4	116	3.18 (1.12)	76.7

*N* number of patients without missing values, *SD* standard deviation

^a^scaling: 0 = not at all important to 4 = very important; ‘does not apply to me’ was equated with ‘not at all important’ for the calculation of means and standard deviations, i.e., percentage calculated with 100% including patients ticking’does not apply’

^b^importance rating

**Table 3:** Descriptive statistics for the Patient Benefits Questionnaire

Item (treatment benefit)		Children				Adolescents			
The current treatment has helped me to …		N	Did apply (%)	Mean (SD)^a^	Quite/ very^b^ (%)	N	Did apply (%)	Mean (SD)^a^	Quite/ very^b^ (%)
1)	… no longer have to sneeze	167	96.5	2.21 (1.22)	43.7	90	95.7	2.18 (1.21)	45.6
2)	… no longer have a runny or stuffy nose	168	97.7	2.19 (1.23)	42.9	92	98.9	2.28 (1.18)	51.1
3)	… be able to breathe through my nose freely	166	97.6	2.23 (1.21)	41.0	91	97.8	2.43 (1.21)	54.9
4)	… feel less tired or groggy	130	75.6	2.35 (1.26)	50.8	75	80.6	2.24 (1.29)	49.3
5)	… be able to stay outdoors without symptoms	155	91.7	2.43 (1.25)	51.0	86	92.5	2.45 (1.20)	51.2
6)	… have an easily applicable treatment	152	89.9	3.25 (1.08)	78.9	82	87.2	2.96 (1.22)	69.5
7)	… not have itching eyes, nose or throat anymore	165	96.5	2.30 (1.24)	48.5	86	92.5	2.37 (1.12)	52.3
8)	… not have burning or watery eyes anymore	148	85.5	2.36 (1.29)	48.0	88	93.6	2.41 (1.28)	54.5
9)	… no longer have hay fever symptoms	168	97.7	2.30 (1.30)	45.8	91	97.8	2.32 (1.20)	48.4
10)	… be able to sleep better	131	76.6	2.59 (1.24)	56.5	73	77.7	2.55 (1.33)	63.0
11)	… need less time for treatment	145	85.3	3.27 (1.21)	79.3	80	86.0	3.08 (1.20)	75.0
12)	… feel well even with having hay fever	156	90.7	2.67 (1.18)	60.3	81	86.2	2.77 (1.22)	70.4
13)	… be able to do anything I want in my free time even while having hay fever	149	86.6	2.83 (1.20)	63.8	77	81.9	2.75 (1.29)	62.3
14)	… feel more comfortable being around other people	123	71.1	2.87 (1.23)	65.9	69	74.2	2.67 (1.31)	63.8
15)	… be focused at school	120	69.8	2.63 (1.25)	62.5	70	74.5	2.49 (1.32)	54.3
16)	… not be excluded by others	83	48.5	2.54 (1.33)	55.4	47	50.0	2.68 (1.42)	61.7
17)	… not to have to go to the doctor so often	142	82.1	2.89 (1.33)	69.0	75	79.8	2.73 (1.37)	68.0
18)	… have a comfortable treatment	157	91.3	3.12 (1.20)	74.5	85	91.4	2.95 (1.23)	68.2
19)	… have fewer side effects	148	86.0	3.03 (1.28)	72.3	81	87.1	2.72 (1.39)	65.4

^a^scaling: 0 = did not at all help to 4 = helped very much; ‘did not apply to me’ was treated as missing value in this analysis, i.e., percentage calculated with 100% excluding patients ticking ‘did not apply’, as for these patients, the goal was not important

^b^benefit rating

### Validation of the PBI-AR-K

#### Population

The sample consisted of 345 patients with a mean age of 11.1 (SD = 3.17; min = 5; max = 17), of which 60.9% (n = 210) were male. The 223 children had a mean age of 9.2 (SD = 2.1; min = 5; max = 12) with a share of 64.6% (n = 144) male participants; the 122 adolescents had a mean age of 14.6 (SD = 1.5; min = 13; max = 17) and a share of 54.1% (n = 66) of males. Allergic rhinitis was present in 98.7% (n = 220) of children and in 100% (n = 122) of adolescents, and allergic conjunctivitis in 82.5% (n = 184) and 77.0% (n = 94), respectively. Allergy to grass pollen was diagnosed in 97.8% (n = 218) of children and 99.2% (n = 121) of adolescents; 59.6% (n = 133) and 63.1% (n = 77) had an allergy to rye or cereal pollen, respectively. Concomitant allergies (Table 1) were present in 64.1% (n = 143) of the children and 64.8% (n = 79) of the adolescents. Concomitant asthma was reported for 39.0% (n = 87) and 27.9% (n = 34) of children and adolescents, respectively.

#### Patient-relevant needs

**Fig. 1:**
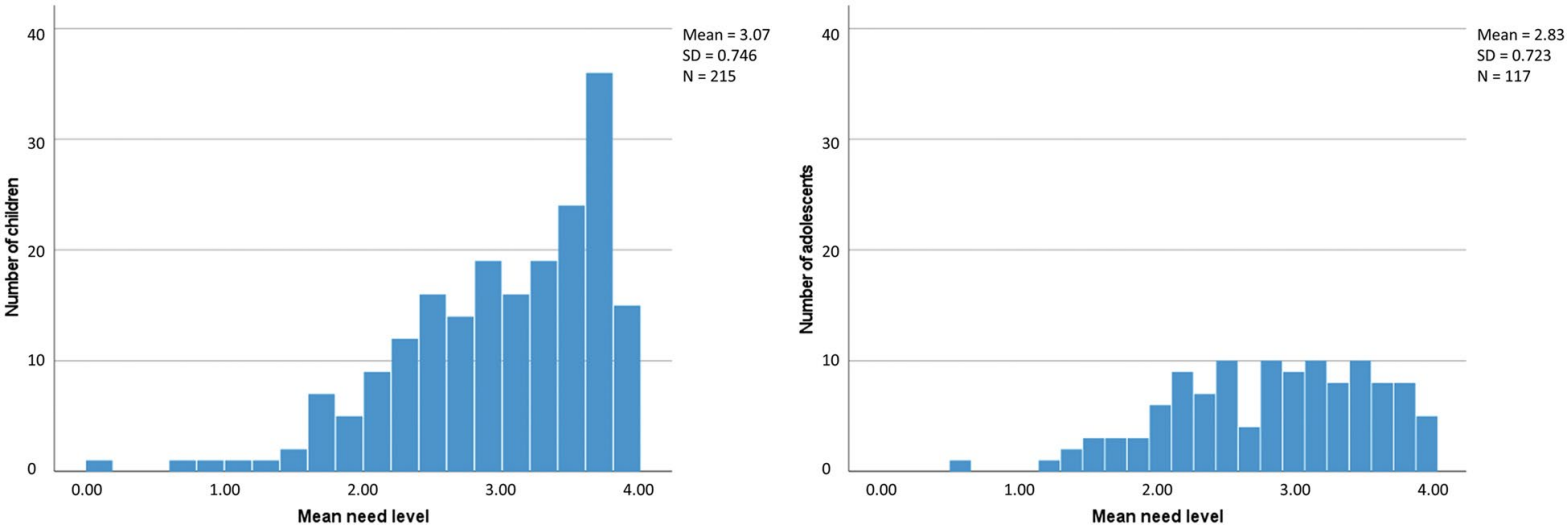
Overall need level in children and adolescents

**Fig. 2:**
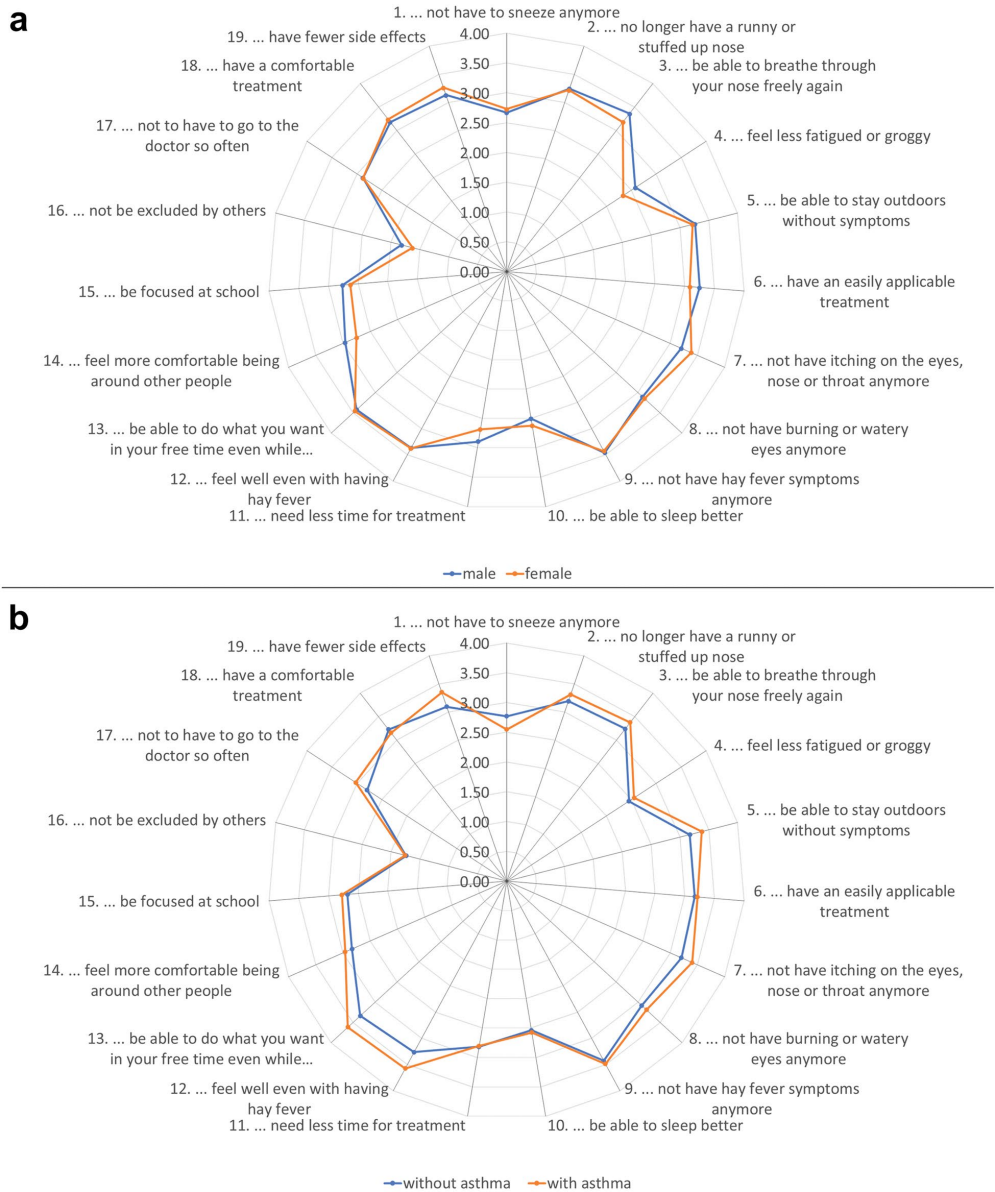
Patient needs as reported by sex (**a**) and by concomitant asthma (**b**) displayed in a spider chart. Dots represent the mean need level per subgroup; the closer the dots are to the outer edge, the higher is the mean value

The items with the highest mean importance ratings were ‘to be able to do anything you want in your free time even while having hay fever’ (mean = 3.5) in children and ‘no longer have hay fever symptoms’ (mean = 3.3) in adolescents (Table 2; see also Klein et al. [24] for a detailed discussion of the results). The item with the lowest importance rating as measured with the PNQ was ‘not be excluded by others’ with a mean score of 1.9 in children and 1.4 in adolescents. Despite this latter item, the mean importance of all other items was 2.5 or higher in children and 2.4 or higher in adolescents. Comparing the mean values of patients’ responses to the need items, children showed a higher need level than adolescents (Fig. 1) indicating higher burden and need for treatment. Similar need patterns were observed for female and male patients as well as for patients with and without asthma (Fig. 2).

#### Patient benefits

**Fig. 3:**
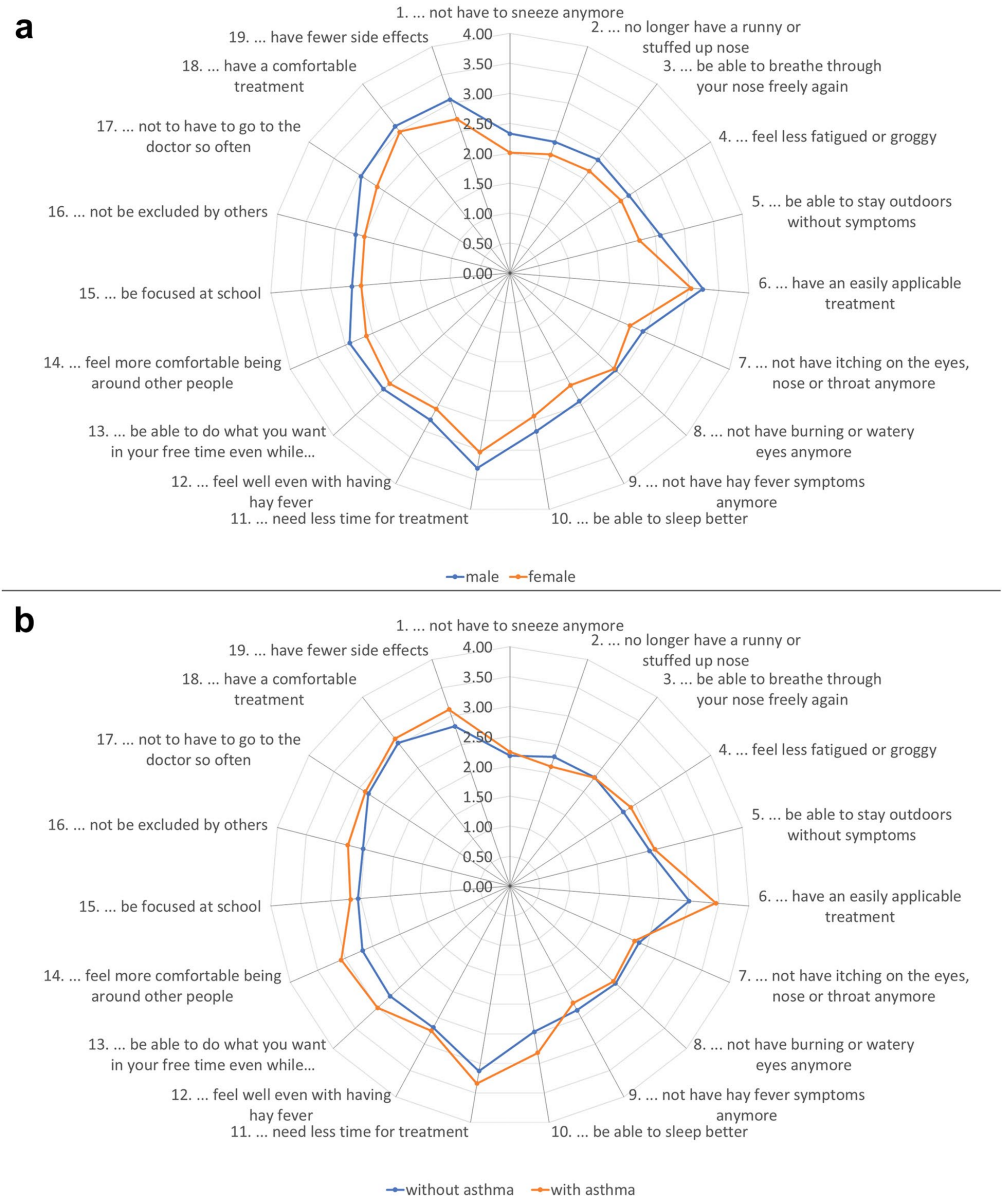
Patient benefit as reported by sex (**a**) and by concomitant asthma (**b**) displayed in a spider chart. Dots represent the mean need level per subgroup; the closer the dots are to the outer edge, the higher is the mean value

The items with the highest patient-reported treatment benefit as measured with the PBQ (Table 3) were ‘need less time for treatment’ (children: 3.3, adolescents: 3.1) and ‘have an easily applicable treatment’ (children: 3.3, adolescents: 3.0). The lowest mean values were found for ‘no longer have a runny or stuffy up nose’ (children: 2.2, adolescents: 2.3), and ‘no longer have to sneeze’ (children and adolescents: 2.2; see also Klein et al. [24] for a detailed discussion of the results). Benefit ratings showed similar patterns by sex and by the prevalence of asthma with somewhat higher benefits for male patients and those with asthma (Fig. 3).

#### Factor analysis

In the children’s sample, factor analysis including data from 204 patients revealed four factors with an eigenvalue > 1, explaining 59.8% of all items’ variance. Based on factor loadings, four meaningful subscales could be defined (Table 4), namely ‘treatment burden’, ‘fatigue/social life‘, ‘physical symptoms‘, and ‘being outdoors‘. Items loading on more than one factor were assigned to the factor on which they had the highest loading.

**Table 4:** Factor loadings and assignment of items to subscales: children

	Factor 1	Factor 2	Factor 3	Factor 4
Items in subscale 1 (‘treatment burden’): Cronbach's alpha = 0.818				
06) … have an easily applicable treatment	**0.698**			
11) … need less time for treatment	**0.799**			
17) … not to have to go to the doctor so often	**0.698**	0.315		
18) … have a comfortable treatment	**0.756**			
19) … have fewer side effects	**0.446**			
Items in subscale 2 (‘fatigue/social life’): Cronbach's alpha = 0.815				
01) … no longer have to sneeze	0.328	**0.369**		
04) … feel less tired or groggy		**0.755**		
10) … be able to sleep better		**0.699**		
14) … feel more comfortable being around other people		**0.608**		0.321
15) … be focused at school	0.366	**0.651**		
16) … not be excluded by others		**0.723**		
Items in subscale 3 (‘physical symptoms’): Cronbach's alpha = 0.813				
02) … no longer have a runny or stuffy nose			**0.780**	
03) … be able to breathe through your nose freely			**0.794**	
09) … no longer have hay fever symptoms			**0.572**	0.382
12) … feel well even with having hay fever			**0.655**	0.348
Items in subscale 4 (‘being outdoors’): Cronbach's alpha = 0.771				
05) … be able to stay outdoors without symptoms			0.314	**0.681**
07) … not have itching on the eyes, nose or throat anymore	0.378			**0.630**
08) … not have burning or watery eyes anymore				**0.793**
13) … be able to do anything you want in your free time even while having hay fever			0.438	**0.596**

N = 204; loadings < 0.3 not shown; in bold: highest loading of the respective item

In the adolescents’ sample, factor analysis including data from 103 patients revealed five factors with an eigenvalue > 1, explaining 67.3% of all items’ variance. After considering the content, three meaningful subscales were defined from this (Table 5), namely ‘treatment burden‘, ‘physical symptoms‘, and ‘psychological burden‘.

**Table 5:** Factor loadings and assignment of items to subscales: adolescents

	Factor1	Factor 2^b^	Factor 3^c^	Factor 4^c^	Factor 5^b^
Items in subscale 1 (‘treatment burden’): Cronbach's alpha = 0.819					
06) have an easily applicable treatment	**0.790**				
11) need less time for treatment	**0.762**				
17) not to have to go to the doctor so often	**0.703**				
18) have a comfortable treatment	**0.802**				
19) have fewer side effects^a^	0.516			**0.541**	
Items in subscale 2 (‘physical symptoms’): Cronbach's alpha = 0.844					
01) no longer have to sneeze		**0.531**			
02) no longer have a runny or stuffy nose		**0.880**			
03) be able to breathe through your nose freely		**0.846**			
05) be able to stay outdoors without symptoms		**0.465**		0.396	0.460
07) not have itching on the eyes, nose or throat anymore					**0.799**
08) not have burning or watery eyes anymore					**0.837**
09) no longer have hay fever symptoms		**0.509**			0.503
Items in subscale 3 (‘psychosocial burden’): Cronbach's alpha = 0.786					
04) feel less tired or groggy		0.368	**0.616**		
10) be able to sleep better		0.385	**0.723**		
12) feel well even with having hay fever	0.303			**0.730**	
13) be able to do anything you want in your free time even while having hay fever				**0.858**	
14) feel more comfortable being around other people			**0.559**	0.527	
15) be focused at school			**0.772**		
16) not be excluded by others			**0.703**		

N = 103; loadings < 0.3 not shown; in bold: highest loading of the respective item

^a^item 19 loaded highly on both factor 1 and factor 4. For content-related reasons, it was assigned to subscale 1 for which the loading was second highest

^b^items with highest loadings on factor 2 or factor 5 were both assigned to subscale 2 for content-related reasons

^c^items with highest loadings on factor 3 or factor 4 were both assigned to subscale 3 for content-related reasons

#### Distribution of global and subscale scores

The mean weighted PBI-AR-K global score was 2.61 (SD = 0.99, median = 2.78) in children and 2.55 (SD = 1.04, median = 2.68) in adolescents with 94.6% and 90.7%, respectively, attaining an at least minimally relevant benefit of 1.0 or higher. In both samples, the subscale ‘treatment burden’ showed the highest mean score with 2.82 (SD = 1.14, median = 3.00) in children and 2.68 (SD = 1.15, median = 2.85) in adolescents. The remaining subscales also showed mean scores above 2.0 in both children (‘fatigue/social life’: 2.08, SD = 1.17, median = 2.08; ‘physical symptoms’: 2.24, SD = 1.13, median = 2.25; ‘being outdoors’: 2.24, SD = 1.16, median = 2.38) and adolescents (‘physical symptoms’: 2.30, SD = 1.06, median = 2.36; ‘psychosocial burden’: 2.35, SD = 1.22, median = 2.55; see also Klein et al. [24] for a detailed discussion of the results).

#### Internal consistency

Global scales and most subscales achieved good internal consistency with Cronbach’s alpha above 0.8 for both children (global: 0.903, ‘treatment burden’: 0.818, ‘fatigue/social life’: 0.815, ‘physical symptoms’: 0.813) and adolescents (global: 0.881, ‘treatment burden’: 0.819, ‘physical symptoms’: 0.844). In both age groups, one subscale showed lower but still acceptable internal consistency: ‘being outdoors’ in children (0.771) and ‘psychosocial burden’ in adolescents (0.786).

#### Convergent validity

Analysis of convergent validity (Table 6) revealed that in children, change in average impairment due to rhinitis symptoms (from visit 1 to visit 2) was significantly correlated with all PBI-AR-K total and subscale scores (r = − 0.22 to r = − 0.44). Change in impaired performance was significantly associated with the global score (d = − 0.51), the subscale ‘physical symptoms’ (d = -0.49), and the subscale ‘being outdoors’ (d = − 0.61). All other convergent variables were significantly associated with the PBI-AR-K global score and the subscales ‘fatigue/social life’, ‘physical symptoms’, and ‘being outdoors’, but not with the subscale ‘treatment burden’.

**Table 6:** Convergent validity of the PBI-AR-K global and subscale sores (in bold: significant correlations)

		Children					Adolescents			
		Global score	Treatment burden	Fatigue/ social life	Physical symptoms	Being outdoors	Global score	Treatment burden	Physical symptoms	Psychosocial burden
Physicians’ global judgement of patients' well-being^†, a/t^	F/t	**26.104**	2.262	**12.056**	**27.07**	**24.109**	**3.329**	1.626	**3.579**	1.757
	p	** < 0.001**	0.076	** < 0.001**	** < 0.001**	** < 0.001**	**0.001**	0.109	**0.001**	0.087
	η^2^/d	0.248	0.029	0.14	0.256	0.237	0.756	0.387	0.813	0.42
	N	161	157	151	160	158	80	77	80	72
Patients’ global judgement of well-being^†, a/t^	F/t	**30.587**	2.28	**14.801**	**28.928**	**23.756**	1.831	- 0.04	**3.139**	0.396
	p	** < 0.001**	0.106	** < 0.001**	** < 0.001**	** < 0.001**	0.071	0.968	**0.002**	0.693
	η^2^/d	0.284	0.03	0.171	0.274	0.239	0.415	- 0.009	0.712	0.095
	N	157	153	147	156	154	78	74	78	69
Changes in rhinitis severity^‡, t^	t	- **3.671**	- 0.712	**-3.792**	**-4.304**	**-3.542**	-1.35	- 0.872	- **2.112**	- 1.28
	p	** < 0.001**	0.477	** < 0.001**	** < 0.001**	**0.001**	0.18	0.386	**0.038**	0.204
	d	- 0.702	- 0.138	-0.747	-0.824	-0.679	-0.383	- 0.249	- 0.6	- 0.39
	N	160	156	150	159	157	87	83	87	77
Change in conjunctivitis severity^‡, t^	t	- **3.556**	- 0.477	- **4.000**	- **4.201**	- **4.06**	- **2.608**	- **2.432**	- **2.904**	- **2.196**
	p	**0.001**	0.634	** < 0.001**	** < 0.001**	** < 0.001**	**0.011**	**0.017**	**0.005**	**0.032**
	d	- 0.693	- 0.095	- 0.724	- 0.82	- 0.793	- 0.73	- 0.702	- 0.813	- 0.676
	N	151	147	143	149	149	79	75	79	69
Change in frequency rhinitis symptoms^‡, t^	t	- **3.028**	- 0.907	- **2.986**	- **3.157**	- **2.844**	- **3.277**	- 1.038	- **2.964**	- **3.91**
	p	**0.003**	0.367	**0.004**	**0.001**	**0.005**	**0.002**	0.303	**0.004**	** < 0.001**
	d	- 0.54	- 0.15	- 0.545	- 0.637	- 0.576	- 1.016	- 0.324	- 0.919	- 1.231
	N	123	120	116	123	120	65	62	65	58
Change in duration rhinitis symptoms^‡, t^	t	- **2.693**	- 1.212	- **3.453**	- **2.849**	- **3.233**	- 1.592	0.178	- 1.667	- **2.739**
	p	**0.008**	0.228	**0.001**	**0.005**	**0.002**	0.116	0.859	0.1	**0.008**
	d	-0.512	- 0.213	- 0.623	- 0.542	- 0.617	- 0.439	0.05	- 0.46	- 0.787
	N	121	117	115	120	119	67	64	67	59
Change in impaired sleep^§, t^	t	- **4.763**	- 1.819	- **3.755**	- **4.300**	- **4.546**	- 1.632	- 1.161	- 1.578	- **2.27**
	p	** < 0.001**	0.072	** < 0.001**	** < 0.001**	** < 0.001**	0.108	0.25	0.12	**0.027**
	d	- 1.011	- 0.392	-0.805	- **0.925**	- 0.968	- 0.617	- 0.439	- 0.579	- 0.863
	N	115	112	109	**114**	113	63	63	63	59
Change in impaired activities^§, t^	t	- **2.862**	- 1.145	- **2.207**	- **3.112**	- **3.067**	- 0.414	0.412	- 0.4	0.412
	p	**0.005**	0.254	**0.029**	**0.002**	**0.003**	0.68	0.682	0.69	0.675
	d	- 0.532	- 0.216	- **0.419**	- **0.576**	- **0.572**	- 0.102	0.102	- 0.098	-0.106
	N	139	135	**131**	**138**	**137**	77	75	77	70
Change in impaired performance^§, t^	t	- **2.108**	- 0.045	- 1.922	- **2.050**	- **2.516**	- 1.032	0.108	- 1.138	- 0.988
	p	**0.037**	0.964	0.058	**0.043**	**0.013**	0.306	0.915	0.26	0.327
	d	- 0.507	- 0.011	- 0.472	-0.493	- 0.605	- 0.291	0.031	- 0.321	- 0.283
	N	103	100	100	103	103	65	63	65	60
Change in average impairment due to rhinitis symptoms^¶, r^	r	- **0.438**	- **0.216**	- **0.337**	- **0.426**	- **0.431**	- **0.424**	- **0.309**	- **0.492**	- **0.318**
	p	** < 0.001**	**0.007**	** < 0.001**	** < 0.001**	** < 0.001**	** < 0.001**	**0.005**	** < 0.001**	**0.005**
	N	159	156	149	158	156	87	83	87	77
Change in peak impairment due to rhinitis symptoms^¶, r^	r	- **0.428**	- 0.108	- **0.364**	- **0.426**	- **0.429**	- **0.304**	- 0.152	- **0.397**	- 0.224
	p	** < 0.001**	0.178	** < 0.001**	** < 0.001**	** < 0.001**	**0.004**	0.173	** < 0.001**	0.051
	N	159	156	149	158	156	86	82	86	75

All variables are physician-reported, except ‘patients’ global judgement of well-being’, ‘change in average/peak impairment due to rhinitis symptoms’

F, F-value (based on analysis of variance [ANOVA]); t, t-value (based on t test); p, p-value (< 0.05 indicating significance); η^2^, eta squared (effect size of ANOVA); d, Cohen’s d (effect size of t-test), N, number of participants

Response options included in this analysis:

^†^children: ‘much better’ / ‘somewhat better’ / ‘unchanged’; adolescents: ‘much better’ / ‘somewhat better’ (response options ‘worse’ (children) and ‘unchanged’ / ‘worse’ (adolescents) were excluded from the analysis for having to few responses)

^‡^ ‘did not decrease’ / ‘did decrease’

^§^ ‘not normalised’ / ‘normalised’

^¶^numeric visual analogue scale: ‘not at all disturbing’ (0) to ‘extremely disturbing’ (10)

Applied tests: ^a^ANOVA (convergent variables with more than two subgroups); ^t^ t test (convergent variable with two subgroups); ^r^ Pearson correlation (continuous convergent variable)

In adolescents, change in average impairment due to rhinitis symptoms (r = − 0.31 to r = − 0.49) and change in conjunctivitis severity (d = − 0.68 to d = 0.81) were significantly associated with all PBI-AR-K scores. Furthermore, change in the frequency of rhinitis symptoms was significantly associated with all scales except the subscale ‘treatment burden’ (d = − 0.91 to d = -1.23). Physicians’ global judgement of patient well-being compared to the previous pollen season as well as change in peak impairment due to rhinitis symptoms were significantly correlated with the global score (η^2^ = 0.76 and r = − 0.30, respectively) and the subscale ‘physical symptoms’ (η^2^ = 0.81 and r = − 0.40, respectively). Besides, the subscale ‘physical symptoms’ showed significant associations with the patients’ global judgement on improvement (η^2^ = 0.71) and change in rhinitis severity (d = − 0.60). Additionally, the subscale ‘psychosocial burden’ showed significant association with change in the duration of rhinitis symptoms (d = − 0.78) and change in impaired sleep (d = − 0.86).

## Discussion

Allergic rhinitis is a frequently prevalent condition especially in younger populations. The use of patient-reported outcomes besides clinical outcomes is decisive to evaluate a treatment comprehensively. As children and adolescents might have a different understanding of specific constructs, and as younger patients have a different use of words than adults, age-specific questionnaires are required to obtain valid and reliable data. Therefore, this article describes the development and validation of the PBI-AR-K, a newly developed version of the PBI-AR for children and adolescents containing 19 items.

Overall, the needs of children and adolescents were similar to those of adult patients with most important needs being to have reduced symptoms and to be able to pursue leisure activities [24]. In children, the overall need level and the number of patients with at least minimally relevant benefit was higher in comparison with adolescents, but also in comparison with adults [24]. According to the algorithm for calculating the Patient Benefit Index score, an elevated need level does not lead to higher benefit scores in the PBI (as the score is divided by the sum of the need items). But from a clinical point of view, higher need levels indicate higher burden to the individual patient which makes treatment necessary and at the same time gives the opportunity to experience treatment benefit even with small symptom reductions. In both children and adolescents, the highest benefit levels were achieved in treatment-related items and, accordingly, in the subscale ‘treatment burden’.

Based on factor analyses, different subscales were defined for children and adolescents. The only equivalent subscale in both subgroups was ‘treatment burden’. Even though dimensions of patient-relevant constructs might be similar across different age groups, the meaning and operationalisation of dimensions can vary [19]. The present study supports this assumption; the different subscale solutions allow for age-appropriate analysis of PBI-AR-K data. However, this reduces the possibility to compare results between different age groups or to analyse patient data longitudinally when patients grow from children to adolescents.

In the children’s sample, convergent validity showed quite consistent results across three out of four subscales (‘fatigue/social life’, ‘physical symptoms’, ‘being outdoors’) and the global scale, all of which were significantly associated with almost all convergent variables. In contrast, the subscale ‘treatment burden’ was significantly correlated only with change in average impairments due to rhinitis symptoms. The low number of significant convergent variables for the treatment-related subscale seems to be plausible, as treatment burden is rather associated with the type of treatment than with the clinical outcomes. The adolescents’ sample showed more inconsistent associations with only change in rhinitis severity showing significant associations with all subscales. Among the subscales, ‘physical symptoms’ was associated with the highest number of convergent variables, which can be explained by the strong focus on symptom-specific convergent variables.

The development of paediatric questionnaires is pivotal to assess meaningful data (e.g., Bullinger et al. [19], Eiser and Morse [20], Rothman et al. [21] and Matza et al. [28]). However, there is ongoing discussion whether children under the age of 12 are able to read and answer self-reported questionnaires appropriately [28]. This issue concerns both our open survey and the validation study. In the open survey, children who felt not fluent enough to read and write, and in the validation study, children under the age of 12 had their parents reading out the questions and noting down their responses. It needs to be acknowledged that this might have affected the response of the participants or parents may even have answered for their children. Such proxy-responses might differ from self-reported answers, but as shown in other questionnaires, agreement between self- and proxy-report is quite good [29, 30], even though results should always be interpreted with caution. Another issue that needs to be mentioned about the development of the questionnaire is the low number of participants in the open survey and the small expert panel. Additionally, no information about their ethnicity was collected, which might have limited the reflection of experiences of ethnic minority groups in the development of the questionnaire. This information was also not covered in the validation study; further studies should investigate whether this aspect impacts on patient’s’ understanding of and responses to the PBI-AR-K.

The validation of the PBI-AR-K was conducted in a study evaluating the benefits of one AIT, which must be considered a limitation as this may reduce the variance of both patient characteristics and possible patient benefits. Due to the real-world design of this study, no control group was implemented, which is why no comparison with another or no treatment is possible. However, as the purpose of this article is to validate the PBI-AR-K questionnaire, this does not impact the informative value of the results.

Since AR is a widespread condition in children and adolescents, the development of age-appropriate tools to assess patient-relevant outcomes is crucial. The results of this study suggest that the newly developed PBI-AR-K is a reliable and valid questionnaire to evaluate treatment needs and benefits of children. For adolescents, results were mixed, suggesting a further need to elaborate in how far the items and subscales of the PBI-AR-K validly reflect AR-specific HRQoL experienced in this age group.

## Data Availability

The data are available on reasonable request from the corresponding author.

## Abbreviations

AIT: Allergen immunotherapy
ANOVA: Analysis of variance
AR: Allergic rhinitis
ARC: Allergic rhinoconjunctivitis
HRQoL: Health-related quality of life
PBi: Patient Benefit Index
PBI-AR: Patient Benefit Index for Allergic Rhinitis
PBI-AR-K: Patient Benefit Index for Allergic Rhinitis for Children and Adolescents
PBQ: Patient Benefit Questionnaire
PNQ: Patient Needs Questionnaire
SLIT: Sublingual immunotherapy
